# The tumor core boost study: A feasibility study of radical dose escalation to the central part of large tumors with an integrated boost in the palliative treatment setting

**DOI:** 10.1007/s00066-022-01976-5

**Published:** 2022-07-20

**Authors:** Olaf Wittenstein, Fabian Krause, Mirko Fischer, Justus Domschikowski, Mirko Nitsche, Christoph Henkenberens, Daniel Habermehl, Juergen Dunst

**Affiliations:** 1 Department of Radiation Oncology, Universitätsklinikum Schleswig-Holstein Campus Kiel, Arnold-Heller-Straße 3, Haus L, 24105 Kiel, Germany; 2 Department of Radiotherapy and Special Oncology, Medical School Hannover, Hannover, Germany; 3 Center for Radiotherapy and Radio-oncology Bremen and Westerstede, 28239 Bremen, Germany; 4 Radprax MVZ, Leimbacher Str. 51a, 42281 Wuppertal, Germany

**Keywords:** Inhomogenous dose distribution, Palliative radiotherapy, Bulky tumor, Hypofractionation, Proof of concept, SBRT

## Abstract

**Purpose:**

For patients with large tumors palliative radiotherapy often is the only local treatment option. To prevent toxicity the administered doses are low. Dose escalation to the tumor could be an option to better smyptom control and prolong local control rates. In this prospective study we used a very pragmatic approach with a simultaneously integrated boost (SIB) to an almost geometrically defined tumor core to achieve this. The primary endpoint was to demonstrate feasibility.

**Method:**

Patients with solid tumors > 4 cm in diameter of different histologies were eligible in this single arm, prospective, multi-institutional clinical feasibility trial with two treatment concepts: 5 × 5 Gy with an integrated boost to the tumor core of 5 × 10 Gy or 10 × 3 Gy with a boost of 10 × 6 Gy. The objective of dose escalation in this study was to deliver a minimum dose of 150% of the prescribed dose to the gross tumor volume (GTV) tumor core and to reach a maximum of at least 200% in the tumor core.

**Results:**

In all, 21 patients at three study sites were recruited between January 2019 and November 2020 and were almost evenly spread (9 to 12) between the two concepts. The treated planning target volumes (PTV) averaged 389.42 cm^3^ (range 49.4–1179.6 cm^3^). The corresponding core volumes were 72.85 cm^3^ on average (range 4.21–338.3 cm^3^). Dose escalation to the tumor core with mean doses of 167.7–207.7% related to the nonboost prescribed isodose led to PTV mean doses of 120.5–163.3%. Treatment delivery and short-term follow-up was successful in all patients.

**Conclusions:**

Palliative radiotherapy with SIB to the tumor core seems to be a feasible and well-tolerated treatment concept for large tumors. The applied high doses of up to 50 Gy in 5 fractions (or 60 Gy in 10 fractions) did not cause unexpected side effects in the 42 day follow-up period. Further research is needed for more information on efficacy and long-term toxicity.

**Supplementary Information:**

The online version of this article (10.1007/s00066-022-01976-5) contains supplementary material, which is available to authorized users.

## Introduction

Patients in a palliative setting are usually treated with the intent of rapid symptom control and low side effects. Therefore, radiotherapy is often the only suitable local treatment option. Typical doses and fractionation schedules are 5 fractions of 4–5 Gy or 10 fractions of 3 Gy, corresponding to a dose equivalent of 40 Gy or less in conventional fractionation [1–7]. However, with these low doses the palliative effect sometimes does not last long enough especially with regard to the increased life-expectancy in many palliative patients due to more effective systemic therapies. Especially, in the case of large tumors with a diameter of more than 4–5 cm, long-term tumor control is low and patients could suffer strong symptoms like pain, dyspnea and mass bleeding even though they have been irradiated. To avoid these situations higher local tumor control rates are needed. Higher radiation doses could probably give patients a benefit as long as the treatment remains safe. As known from other clinical settings, radiation doses of 100 Gy biologically equivalent total dose at 2 Gy/fraction (EQD2) or more achieve local tumor control rates of 80–90% even in relatively radioresistant tumors [8, 9]. Therefore, optimization of mean dose to the gross tumor volume (GTV) is crucial [10]. Safe delivery of such high doses using stereotactic body radiotherapy (SBRT) is possible and has emerged to a standard-of-care-procedure in (locally) curative treatment of inoperable T1–2 lung cancers or oligometastases [11, 12]. However, stereotactic techniques have some limitations, mainly the essential need for a steep dose-gradient to spare the surrounding organs at risk (OAR) [13]. The required steep dose gradient which depends on the size of the target volume can be easily achieved in small tumors. In larger tumors, safe dose escalation is a clinical challenge. A homogeneous dose escalation to the whole gross tumor volume (GTV) is not possible without increased risk for side effects. A theoretical concept for better local tumor control while maintaining tolerability could be an increase in mean tumor dose with dose escalation only to the central part of the GTV using a simultaneously integrated boost (SIB). These higher doses to the central and possibly hypoxic and radioresistant part could achieve an additional benefit in local tumor control [14, 15].

Palliative patients with large tumors often show symptoms like shortness of breath or pain. These patients are not good candidates for high-precision techniques which require either strict immobilization or are time-consuming; thus, the inclusion of a SIB for dose escalation should require standard techniques with robust plans. Whether optimization and administration of such plans in a wide spectrum of clinical situations is possible requires further investigation of optimal concepts.

We report the results of a prospective feasibility study in which robust volumetric modulated arc therapy (VMAT) plans in standard settings were used to achieve the announced dose escalation in the palliative treatment of patients with large (> 4 cm) tumors in different clinical situations.

## Methods and materials

### Study design

The tumor core boost study was a single arm, prospective, multi-institutional clinical feasibility trial with a planned sample size of 25 patients treated with palliative intent for tumors (primary or metastases) larger than 4 cm in diameter. Treatment consisted of palliative radiotherapy with 30 Gy in 10 fractions or 25 Gy in 5 fractions. Which regimen to use was the investigator’s choice and depended on the general assessment of the patient’s overall clinical situation. The center of the target volume (the “tumor core”) received an additional boost dose and the dose to the core was escalated by 100% as compared to the standard prescription dose; thus, the integrated boost volume received a total dose of 60 Gy in 10 fractions or 50 Gy in 5 fractions. The aim of the study was to demonstrate that dose escalation with this SIB concept is feasible in a non-SBRT setting without high rates of side effects because of a significantly increased dose to surrounding organs at risk.

The study complied with the Declaration of Helsinki, and the protocol was approved by ethics committee of the Christian-Albrechts-University Kiel (D535/18) and listed on German Clinical Trials Register (DRKS00015763). As decided by DEGRO Expert Panel (inquiry 116), no approval analog §23 *Strahlenschutzverordnung* or §28a *Röntgenverordnung* was needed.

### Inclusion and exclusion criteria

Patients were eligible if they had a large solid tumor (≥ 4 cm in diameter), almost irrespective of histology (lymphoma and other hematological malignancies were excluded), and were scheduled for palliative radiotherapy to this lesion. Further inclusion criteria were age above 18 years and informed consent. Moreover, a tumor core of at least 2 cm in spherical diameter with a 1 cm margin in all directions inside the GTV was to be definable.

Patients were excluded if critical normal tissue structures (CNTS) were inside the GTV (e.g., large vessels, main bronchi) to avoid major complications due to overdosage to such critical volumes. Moreover, patients were excluded in case of pregnancy, high grade renal failure (glomerular filtration rate < 30 ml/min), and legal incapacity.

### Contouring of target volumes

The GTV included the whole macroscopic tumor as defined in the planning computed tomography (CT). Additional information from other imaging modalities like magnetic resonance imaging (MRI) or positron emission tomography CT (PET/CT) was also used, if available. The SIB volume, called GTV tumor core, was defined as a rotund volume measuring at least 20 mm in diameter and lying completely inside the GTV with an additional inner margin of at least 10 mm in all directions; this inner margin was chosen to safely avoid overdosage outside the GTV even in case of relatively larger motion during treatment. Further information on tumor biology (e.g., amount of Fluordesoxyglucose (FDG) uptake in PET scanning or visible necrosis) was not considered for target volume definition in this study. Other macroscopic manifestations of tumor-like nodal metastases measuring < 4 cm could be included in the clinical target volume (CTV), but not in the high-dose GTV tumor core. The additional margin for the planning target volume (PTV) depended on the internal standards of the participating institutions.

### Organ-at-risk management

The listed OARs were to be contoured if they were within 5 cm in cranial-to-caudal orientation of the GTV: brain, pituitary gland, optic nerves, optic chiasm, eyes, lenses, parotid glands, spinal cord, lungs, heart, esophagus, liver, kidneys, bladder, rectum, bowel bag. For every OAR of every patient, mean dose (D_mean_) and max dose (D_max_) were documented and compared with the doses of an additional standard radiotherapy (RT) plan with homogenous dose distribution without SIB. To simplify this plan comparison of the different OARs with the different fractionation regimes, we defined new patient-specific parameters: the maximum increase of any OAR’s D_mean_ and D_max_ calculated in Gy and %.

### Treatment planning, documentation, and application

The prescribed dose was the dose on the PTV-surrounding isodose. The objective of dose escalation in this study was to deliver a minimum dose of 150% of the prescribed dose to the GTV tumor core and to reach a maximum of at least 200% in the tumor core. D_mean_, minimum dose (D_min_), and D_max_ to the PTV and GTV tumor core were documented. In addition, a standard plan with homogeneous dose distribution must be calculated for all patients; this could be, depending on the clinical situation, an appropriate VMAT, intensity modulated RT (IMRT) or a 3-dimensional conformal RT plan (3D-CRT).

Radiation was to be applied with daily image guidance via cone beam CT (CBCT) or stereoscopic X‑rays, if possible. Due to the palliative setting, exceptions were allowed for patients with special impairments. Overall treatment time (from the first to the last treatment fraction) should not exceed 3 weeks for patients receiving 10 fractions and 2 weeks for patients with 5 fractions.

### Tolerability and follow-up

Patients were clinically investigated at baseline before start of radiation treatment (d0), weekly during radiotherapy at treatment days d1, d7, d14, and at the day of last treatment (RT+0d). Early follow-up visits at day RT+7d and day RT+14d were done via phone; at day RT+42d, all patients were clinically investigated. Because of the estimated short overall survival of the patients and the short follow-up period with focus on feasibility, an investigation of tumor response (RECIST criteria) was not performed.

### Endpoints

The primary endpoint of the study was feasibility of a SIB with 100% dose escalation to the tumor core in palliative RT. Feasibility criteria were defined as

- Optimization of treatment plans with SIB with protocol-defined maximum doses to the tumor core and
- Successful administration of the planned RT dose in at least 80% of patients.

Secondary endpoints were toxicity and side-effects of radiotherapy with SIB. Side effects and SAEs (serious adverse event) were documented according to the National Cancer Institute Common Terminology Criteria for Adverse Events (NCI CTCAE) v4.03 [16]. It was hypothesized that less than 20% of the treated patients would show acute toxicities of grade ≥ 2 during treatment and within 42 days after.

### Patients’ characteristics

Recruitment of patients was lower than expected. We included a total of 21 patients from January 2019 until November 2020 until the study was closed at the end of 2020. All patients gave their informed consent. The patients were almost evenly spread over both fractionation regimes. Treated locations differed a lot. In 7 of 21 cases, tumors were located in the head and neck region, in 5 cases in the thoracic region (three intrapulmonal, two extrapulmonal), and in 9 cases the tumors were located in the abdomen or pelvis. In all, 7 primary tumors, 5 nodal metastases, and 10 distant metastases were treated. Non-small cell lung cancer (NSCLC) was the most common histology (8 of 21 patients, 38.1%). Other histologies included endometrial cancer, head and neck squamous cell carcinoma (HNSCC), rectal cancer, melanoma, breast cancer, fallopian tube cancer, sarcoma, and squamous cell carcinoma of unknown primary (SSC-CUP). More patient characteristics are listed in Table 1.

**Table 1:** Patient characteristics before treatment planning

Characteristics		All patients, *N*	Patients treated with 5 fractions of 5/10 Gy, *N*	Patients treated with 10 fractions of 3/6 Gy, *N*
Patients	*N*	21	9	12
Sex	Male	13	6	7
	Female	8	3	5
Age (years)	50–60	8	3	5
	61–70	4	2	2
	71–80	6	2	4
	81–91	3	2	1
ECOG status	0–1	14	8	6
	2–3	7	1	6
Histology	NSCLC	8	4	4
	SCC-CUP	2	1	1
	HNSCC	2	1	1
	Sarcoma	3	1	2
	Other	6	2	4
Treated location	Head and neck	7	3	4
	Thorax	5	2	3
	Abdomen/Pelvis	9	4	5
Largest tumor diameter	4–8 cm	13	3	10
	8–12 cm	6	4	2
	12–16 cm	2	2	0

*ECOG* Eastern Cooperative Oncology Group, *NSCLC* non-small cell lung cancer, *SCC-CUP* squamous cell carcinoma of unknown primary, *HNSCC* head and neck squamous cell cancer

## Results

### Treatment adherence

The prescribed treatment was successfully delivered in all patients. More than 95% of the patients (20 of 21) completed treatment with SIB to the tumor core in the scheduled treatment time (10-fractionation regime 11–17 days, 5‑fractionation regime 7–13 days). Treatment of one patient in 5‑fractionation regime was completed within 17 days. No patient’s treatment was abandoned.

### Acute toxicity and adverse events

Rates of acute toxicities at any point of the monitored period were 100% grade ≥ 1, 33.3% grade ≥ 2, and 9.5% grade ≥ 3, respectively. No grade 4 toxicities were observed. Corrected for toxicities documented at d0 or d1 (e.g., caused by prior treatments like chemotherapy or radiotherapy of a different site), the estimated rate of acute toxicities grade ≥ 2 was 28.6%. Considering only those cases where the toxicities lasted until the last follow-up time-point (RT+42d), the rates were 61.1% for toxicities of grade ≥ 1 and 5.6% grade ≥ 2 (Table 2).

**Table 2:** Number of documented toxicities; *n* = 21 until last radiotherapy (RT), *n* = 20 for RT+7 and RT+14, *n* = 18 for RT+42

	Baseline					D1					D7					Last RT					RT+7d					RT+14d					RT+42d				
	0°	1°	2°	3°	4°	0°	1°	2°	3°	4°	0°	1°	2°	3°	4°	0°	1°	2°	3°	4°	0°	1°	2°	3°	4°	0°	1°	2°	3°	4°	0°	1°	2°	3°	4°
Nausea	17	2	1	1		17	1	3			16	3	2			17	3	1			16	4				17	3				18				
Vomiting	18	3				19	2				19	2				19	2				18	2				20					18				
Vertigo	15	5	1			15	6				19	2				18	3				17	3				17	3				15	3			
Stomatitis	19	2				17	3		1		19	1	1			17	3	1			17	3				16	4				15	3			
Esophagitis	21					20	1				20		1			20	1				17	2	1			17	2	1			15	3			
Cough	18	3				15	6				16	4	1			18	3				16	3	1			18	2				17		1		
Dyspnea	17	3	1			16	4	1			16	5				16	4	1			16	4				17	3				17			1	
Radiodermatitis	21					20	1				19	2				18	3				17	2	1			18	1	1			15	3			
Fever	21					21					20	1				21					19	1				18	2				18				
Diarrhea	20	1				19	2				20	1				21					19	1				19	1				18				
Pneumonitis	21					21					21					21					19	1				19	1				18				
Bleeding	18	2	1			19	2				20	1				20	1				18	2				19	1				17	1			

Six adverse events (AE) and three severe adverse events (SAE) were reported. Two SAEs resulted from unplanned hospitalization due to a systemic tumor progression and both patients died in the hospital; there was no evidence for radiation-related side effects. The third SAE was related to death of a patient during palliative care in a hospice. Before death, 2 of the 3 patients showed maximum documented acute toxicity grades of 1, while 1 patient showed grade 2. The reported AEs were moderate pneumonia (treated with infusional antibiotic therapy), moderate local inflammation in the irradiated region (treated with dexamethasone orally), pancytopenia after chemotherapy requiring blood transfusion and granulocyte colony stimulating factor (G‑CSF), serious hemorrhage of a tumor before start of radiation treatment (requiring surgical hemostasis), moderate colitis after immunotherapy (treated with prednisolone), and repeated cardiac decompensations caused by known heart failure with delay of radiation treatment.

### Primary and secondary endpoints

The postulated feasibility requirements, namely the calculation of robust treatment plans with sparing of OARs and successful delivery of the prescribed treatment in more than 80% of the patients, were both met (treatment adherence 100%). With regard to the secondary endpoints, the appearance of toxicities greater than grade 2 on CTCAE scale was 28.6% and thus higher than the expected rate of 20%. However, the vast majority of the observed toxicity derived not from radiotherapy treatment but was rather caused by tumor-specific complications or related to other cancer-specific treatment, mainly chemotherapy.

### Treatment planning and quality of treatment plans

The objectives of dose escalation were a minimum dose to GTV tumor core of 150% (45 Gy in group A, 37.5 Gy in group B) and a maximum dose of at least 200% of the prescript PTV enclosing isodose. These study objectives were reached in more than 90% of the patients. In two cases, minor protocol deviations with a slight underdosage of GTV tumor core were detected.

In the main testing center (study site a, Kiel, Germany) VMAT plans were generated with the treatment planning system Eclipse v.13.7.14 (Varian Medical Systems, Palo Alto, CA, USA) and the associated AAA algorithm for a TrueBeam STx linear accelerator (LINAC). VMAT techniques were used in all patients. In comparison with other modern technologies available in radiotherapy (e.g., fixed field intensity modulated radiotherapy), VMAT is known to be more monitor unit (MU) efficient [17–19]. Due to the dose escalation in the tumor core and the resulting high number of MUs, the RapidArc (RA) technique was used for irradiation. To avoid additional exposure caused by photoneutrons [20] RA plans were generated with 6 MV photon beams for the prescribed dose in the tumor core.

At least half coplanar double arcs were used for all these plans. While using additional ‘shell’ structures, it was possible to modulate the dose gradient within the target volume. The aim was a preferably uniform dose fall from the integrated boost area in the core of the tumor to the target volume enclosing isodose. The number of shells used was based on the size and shape of the target volume.

Normalization was performed typically on the maximum dose value, which was 50 Gy or 60 Gy, respectively, at the target maximum (plan normalization 200% at target maximum). To achieve a higher mean dose in the tumor core, it was also possible to normalize to the tumor core target mean (plan normalization 200% at target mean). For the different normalization methods, the variability of several dose parameters (e.g., dose characteristics of GTV tumor core and PTV; Tab. A1 supplement) were then consecutively analyzed.

**Fig. 1:**
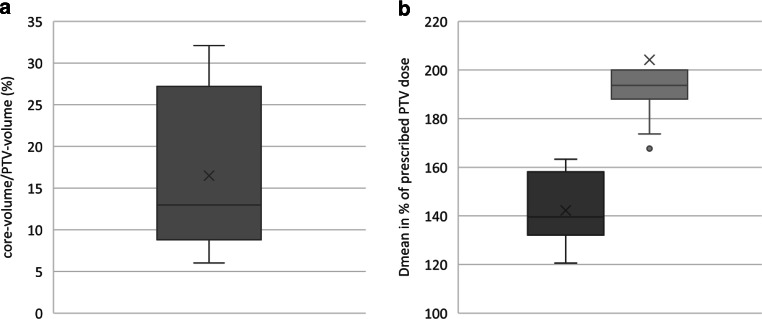
**a** Boxplot of the relative size of the tumor core volume in percentage of the corresponding planning tumor volume (PTV) for all patients. **b** Boxplot of the relative mean doses of PTV (*dark gray*) and core volume (*light gray*) in % of the prescribed PTV dose (30 Gy for group A, 25 Gy for group B); *n* = 21 for both diagrams

The treated PTVs had a mean of 389.42 cm^3^ (range 49.4–1179.6 cm^3^). This conformed to a mean idealized spherical diameter of 9.06 cm. The corresponding core volumes averaged 72.85 cm^3^ (range 4.21–338.3 cm^3^, mean spherical diameter 5.18 cm). The relation of core volume and PTV for all patients is shown in Fig. 1a, while the size of the core volume related to the PTV ranged from 6.0–32.1% (mean 16.5%; Fig. 1b).

**Fig. 2:**
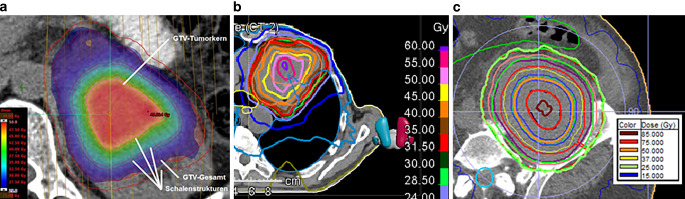
Depiction of dose distribution in axial view of three different tumor core boost plans at the three different study sites with three different planning platforms: **a** Eclipse (Varian Medical Systems, Palo Alto, CA, USA), **b** RayStation (RaySearch, Stockholm, Sweden), **c** Monaco (Elekta, Stockholm, Sweden )

Maximum doses to GTV tumor core and PTV were in all cases identical (in all cases, the GTV tumor core was totally encompassed by the PTV). At the main test center Kiel and in study site b (Bremen, Germany, using RayStation 8.0.1.10, RaySearch, Stockholm, Sweden), plans show a switch from maximum doses of 200% of the prescribed PTV dose to mean doses of exactly 200%. This resulted from the above-mentioned differences in plan normalization and led to higher maximum doses. In study site c (Hannover, Germany), a third approach was used with significantly higher doses than at the other sites. The participating physicists focused on dose escalation as high as possible. First, they optimized the VMAT plans for around 200% dose maximum. Second, the resulting OAR doses were used and increased to obtain a higher dose maximum inside the GTV tumor core, while maintaining the prescript PTV surrounding isodose level. Treatment planning was performed with Monaco 5.10 (Elekta, Stockholm, Sweden). The different planning approaches are illustrated in Fig. 2.

### OAR handling

The benefit of escalated PTV doses had one drawback—a significant increase of doses delivered to surrounding OARs. Altogether dose statistics of 136 delineated OARs were measured. Because the treated sites were located throughout the human body, not all to-be-documented OARs were delineated in every treatment plan. The spinal cord was the most common (delineated in 18 of 21 patients), optic chiasm the least (1 of 21). Doses to OARs in the tumor core boost plan were compared to doses in standard RT plan (without core boost).

**Fig. 3:**
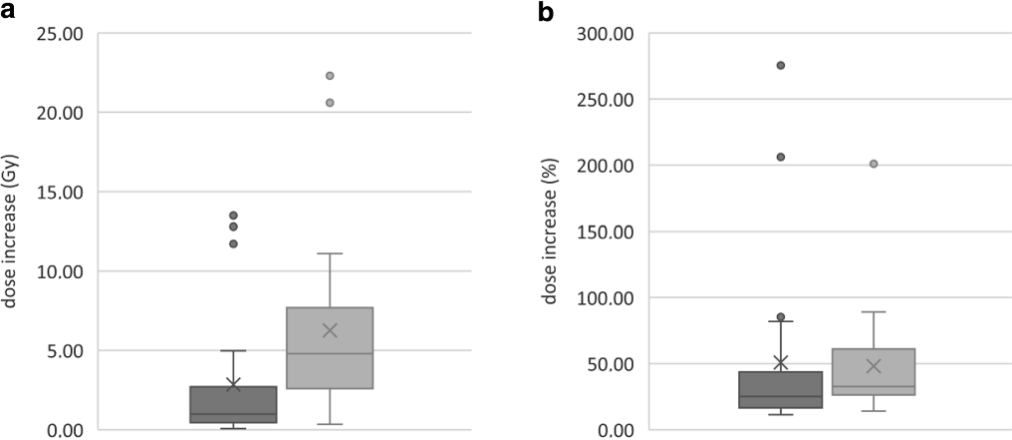
Boxplots of increased doses to organs at risk (OARs) in tumor core boost plans compared to nonboost plans. For each patient, we plotted only data of the OARs which had the highest dose-increase in the four categories. Categories were the increase of Dmean (*dark grey*) and Dmax (*light grey*) in Gy (**a**) and % (**b**). We made no regulations for maximum OAR doses in the study protocol. Thus, the decision for treatment with these increased OAR doses was in the hand of the treating physicians in each study site; *n* = 21 for both diagrams

As an example, patient 21 (Tab. A1 supplement) with a PTV Dmax of 100.5 Gy was selected. Dmax to OAR spinal cord was 33.4 Gy in the core boost treatment plan and 11.1 Gy in the standard plan (both 10 fractions). This was an increase of 22.3 Gy or 200.9%. For another OAR (bowel bag) Dmax was 46.1 Gy (core boost), and 24.6 Gy (conventional). This was an increase of 21.5 Gy or 87.4%. Both parameters were higher for OAR spinal cord than for bowel bag and higher than for any other OAR of this patient. Consequently, 22.3 Gy and 200.9% were recorded for patient 21. Results of all patients are depicted in Fig. 3.

## Discussion

To our knowledge, an integrated boost to partial parts of large GTVs with the objective of radical dose escalation has not yet been investigated in prospective clinical trials although some case reports have described this approach [21, 22]. The latest published results of another radical dose-escalation concept (spatial fractionated SBRT, Lattice) are of retrospective nature [23]. Thus, our clinical results using high doses in large tumors could facilitate implementation of further dose-escalation concepts.

The primary endpoint of this feasibility study, a combination of technical planning criteria and treatment adherence, was met. Creation of treatment plans according to the protocol was possible in all patients and treatment adherence was excellent. Acute toxicity grade ≥ 3 was 28.6% and therefore slightly higher than the estimated 20% but none of the treated patients experienced radiotherapy-related severe toxicity; all severe adverse events were related to the underlying disease and were caused by tumor progression. Thus, this concept of inhomogeneous dose prescription to the GTV with a simultaneous integrated boost to the tumor core was feasible and well tolerated and might offer a tool for dose escalation in palliative treatment of large tumors.

The study has on the other hand disclosed a variety of difficulties with this approach. Although the predefined protocol-specific dose-escalation concept could be adopted in all patients, plan statistics differed a lot. We achieved ablative radiation doses in the center of the GTV with significant increase in PTV mean dose (20–63%), even though sizes of the boost volumes were only 6–31% of the whole PTVs. As expected, relatively larger core volumes led to a higher increase of PTV mean dose. Due to the fact that the inner margin around the core boost volume was set to 1 cm for safety reasons (with an additional 5 mm PTV margin), the boost volume in small tumors was relatively smaller compared to larger ones. To overcome this problem, smaller safety margins would be required for smaller tumors to increase the core volume to the relatively same size as in larger tumors. For example, in a spherical tumor with 5 cm in diameter, the core boost volume makes up only 22% of the whole GTV in case of a safety margin of 1 cm around the core. An increase of the core volume to more than half of the GTV would require reducing the safety margin to about 4 mm. Smaller margins like this require more precise image guidance (as used in SBRT) than were specified in this study [24].

Finally, the individual physical treatment planning also influences plan quality and the level of dose escalation a lot. The used and established techniques of the different planning systems (i.e., VMAT, RapidArc, Dynamic Conformal Arc Therapy [DCAT] and Multi-Criteria-Optimization [MCO]) allow complex dose distributions. Each of the three planning systems used in this study had its advantages and the planning physicists had different ideas about how to deal with the study requirements and we did no central quality assessment of the different treatment plans. Study sites were responsible for their approaches. The use of additional shell structures in study site a might offer more influence in the arrangement of dose hotspots than the stricter dose-maximizing approach in study site c (Fig. 2). The key factor is the weighting between dose-to-tumor and OAR doses. To minimize side effects, doses to OARs in palliative care are kept low. Within the core boost study, these low doses were elevated significantly by 50% and more (up to 275% depicting the one OAR with the highest increase per treatment plan). Doubling a low dose (i.e., 4 Gy instead of 2 Gy D_mean_) might have no clinical effect in palliative situation and is consequently tolerable. On the other hand, one must be very careful with high absolute dose increases such as the outliers in Fig. 3a. An increase of 13.5 Gy in Dmean or 22.3 Gy in D_max_ in 10 daily fractions compared to a well-established treatment regime (10 × 3 Gy) could lead to unpredictable toxicity. These extreme outliers were a consequence of the dose-maximizing approach of study center c. Another crucial impact to OAR statistics was the location of the treated tumor. Chan et al. had shown that dose escalation to lung tumors with an inhomogeneous dose distribution has no significant impact on the mean dose to the lung [25]. Subgroup analysis of five tumors, which were located inside the lungs (metastases or primary tumors) had similar results: the escalation of the mean dose to the lung was low (1.02 Gy, range 0.151–1.40 Gy). Thus, massive dose-escalation to lung tumors with tumor core boost is safe. Other subgroups had higher dose escalation, i.e., the subgroup of four head and neck tumors with increase of Dmean of the ipsilateral parotid gland by a mean of 4.0 Gy (range 1.1–11.7 Gy).

For more precise guidelines for treatment of large tumors, further studies are needed. To overcome the shortcomings of this study (different radiated regions, short follow-up period, no control of tumor response, no strict rules for OAR handling, flexibility in treatment planning), an investigation with a much more homogenous study cohort (i.e., inclusion of bulky lung tumors only) or different set-ups for different tumor sizes and locations is needed. In addition, more precise guidelines for GTV/PTV ratio, dose coverage, and dose gradients should be included. Finally, to validate clinical benefits of the concept different endpoints, more cases and consequently more participating institutes are required. Therefore, an additional multicenter study to implement the SIB concept in the treatment of large tumors is under development.

## Conclusions

Radical dose escalation with more than 50 Gy in 5 fractions or 60 Gy in 10 fractions to the tumor core was feasible in the small subgroup of palliative patients with large tumors. The delivery of the demonstrated SIB technique remains safe, and with use of large safety margins it is simple to implement in daily routine with standard treatment settings. Efficacy needs to be investigated in an additional trial.

## Supplementary Information

Table A1 in the supplementary material shows detailed characteristics of GTV tumor core and PTV for all patients treated with tumor core boost. The patient count is not chronological, instead it is sorted by treatment groups (A, B) and study sites (a, b, c). Deviations to protocol are marked fat.
