# Supplementary material for: The tumor core boost study: A feasibility study of radical dose escalation to the central part of large tumors with an integrated boost in the palliative treatment setting
Source: Strahlenther Onkol. 2022 Jul 20;199(3):258–67. doi: 10.1007/s00066-022-01976-5 (PMC9938025; doi:10.1007/s00066-022-01976-5)
Supplement: Supplementary file 1 — Table A1 in the supplementary material shows detailed characteristics of GTV tumor core and PTV for all patients treated with tumor core boost. The patient count is not chronological, instead it is sorted by treatment groups (A, B) and study sites (a, b, c). Deviations to protocol are marked fat. [file 66_2022_1976_MOESM1_ESM.pdf]

Supplementary Material

Strahlentherapie und Onkologie

The tumor-core-boost study: A feasibility study of radical dose-escalation to the central part of large tumors with an integrated boost in the palliative treatment setting

Olaf Wittenstein, Fabian Krause, Mirko Fischer, Justus Domschikowski, Mirko Nitsche, Christoph Henkenberens, Daniel Habermehl and Juergen Dunst

Corresponding Author: Olaf Wittenstein, Email: olaf.wittenstein@uksh.de
ORCID: 0000-0003-4127-3139

Table A1

Patient No.	*Trea-tment group*	*Study site*	*Prescribed PTV dose /Gy*	*Boost-dose (200%) /Gy*	*GTV-tumor-core*				*PTV*			
					Volume /cm³	*Dmean /Gy*	*Dmin /Gy*	*Dmax /Gy*	*Volume /cm³*	*Dmean /Gy*	*Dmin /Gy*	*Dmax /Gy*
1	*B*	*a*	*5 x 5 = 25*	*50*	58.1	50	44.1	52.3	813.1	33.2	21.4	52.3
2	*B*	*a*	*5 x 5 = 25*	*50*	4.21	48.03	42.54	50	49.4	36.96	13.61	50
3	*B*	*a*	*5 x 5 = 25*	*50*	53.5	47.68	41.88	50	315	35.28	23.53	50
4	*B*	*a*	*5 x 5 = 25*	*50*	42.27	47.2	40.36	50	494.6	30.14	21.86	50
5	*B*	*a*	*5 x 5 = 25*	*50*	4.75	48.42	44.49	50	53	36.16	24.56	50
6	*B*	*a*	*5 x 5 = 25*	*50*	14.1	50	39.38	52.25	160.37	34.52	17.82	52.25
7	*B*	*a*	*5 x 5 = 25*	*50*	129.87	50	43.62	52.8	420.69	39.52	25.39	52.78
8	*B*	*a*	*5 x 5 = 25*	*50*	338,3	50	41.10	53.26	1179.6	39.55	26.56	53.26
9	*B*	*c*	*5 x 5 = 25*	*50*	*361*	86	71	88.8	*1411*	40.75	21	88.8
10	*A*	*a*	*10 x 3 = 30*	*60*	12.67	60	51.7	64.1	142.5	36.9	27.9	64.1
11	*A*	*a*	*10 x 3 = 30*	*60*	8.91	55.98	46.99	60	89.6	37.46	0	*60*
12	*A*	*a*	*10 x 3 = 30*	*60*	20.2	58.1	54.1	60	335	41.4	32.7	60
13	*A*	*a*	*10 x 3 = 30*	*60*	12.97	56.17	49.12	60	40.4	47.95	35.67	60
14	*A*	*a*	*10 x 3 = 30*	*60*	45.9	56.72	47.08	60	168.8	41.86	26.19	60
15	*A*	*a*	*10 x 3 = 30*	*60*	40.53	55.29	47.09	60	186.8	41.09	25.62	60
16	*A*	*a*	*10 x 3 = 30*	*60*	39.521	56.63	**20.92**	60	305.1	39.47	20.92	60
17	*A*	*a*	*10 x 3 = 30*	*60*	97.15	60	52.33	63.41	473.09	43.81	31.52	63.41
18	*A*	*b*	*10 x 3 = 30*	*60*	56.12	52.09	45.45	60.42	432.6	39.77	27.96	60.42
19	*A*	*b*	*10 x 3 = 30*	*60*	115.09	50.31	**44.02**	**59.89**	785.71	37.39	28.09	59.89
20	*A*	*b*	*10 x 3 = 30*	*60*	*48.03*	60	56.5	61.57	171.54	48.63	28.15	61.57
21	*A*	*c*	*10 x 3 = 30*	*60*	*26.6*	92.3	79.7	100.5	150	49	20.8	100.5

Supplement Table A1 - plan characteristics of GTV-tumor-core and PTV for all patients treated with tumor-core-boost. Patient count is not chronological. Instead, it is sorted by treatment groups (A, B) and study sites (a, b, c). Deviations to protocol are marked fat.
